# Macro-engagement in mHealth: Exploring user engagement beyond the screen

**DOI:** 10.1177/20552076231225591

**Published:** 2024-01-23

**Authors:** Camila Villegas Mejía, Danielle Remmerswaal, Rutger C.M.E. Engels, Geke D.S. Ludden, Marilisa Boffo

**Affiliations:** 1 Erasmus School of Social and Behavioural Sciences, 6984 Erasmus University Rotterdam, Netherlands; 2 Faculty of Engineering Technology, University of Twente, Netherlands

**Keywords:** Digital health general, mental health psychology, well-being psychology, engagement, qualitative studies, mHealth psychology, macro-engagement, dis-engagement‌

## Abstract

While digital technology holds great promise for health and well-being, some users feel sceptical about the time they spend online and how they use their mobile devices. This attitude could hamper uptake of digital health technologies and engagement with them. This study uses the concept of macro-engagement as a starting point to investigate how users of digital behaviour change interventions (DBCIs) engage with their behaviour change goals beyond the screens of their tools. Thirty semi-structured interviews were conducted with individuals who take part in behaviour change processes in different ways (i.e. mental health professionals, digital health experts and users of DBCIs). A qualitative analysis of their data through a grounded theory approach highlighted a wide array of offscreen behaviors and strategies that complement a behavior change process offscreen. Furthermore, implications for designing technology that encourages progressive non-reliance on DBCI usage are drawn out.

## Introduction

### Background

With the advent of the internet and mobile technology, digital behaviour change interventions (DBCIs) have been proposed as a promising solution to deliver cost-effective, scalable and accessible tools for users looking to adopt healthier lifestyles.^1–3^ However, though digital interventions hold great promise, they tend to have low engagement rates, which has been theorised might hinder their effectiveness.^2–6^ Simply put, individuals need to use the interventions to benefit from them. As a result, many of the design strategies employed for engagement with digital interventions emulate commercial app engagement strategies geared towards promoting increased usage, for example, notifications, reward tiers and streaks.^7^

Simultaneously, a growing concern surrounds the impact of technology on well-being. Empirical evidence suggests that there are links between screen time and reduced quality of social connections, sedentary behaviour, depressive symptoms, and sleep deprivation and quality.^8^ While findings suggest a negative impact of technology on well-being, they still come with some nuances: not all screen time is equally harmful, not all of it constitutes problematic use and not everyone is affected in the same way.^8,9^ Regardless of nuances and effect sizes, the perception of screens as a negative influence on well-being is a challenge that the digital health field must contend with. When technology is viewed negatively, individuals may be less inclined to engage with it and, consequently, miss out on its potential benefits.^10^

These two notions highlight a paradox within the realm of digital intervention design. On one hand, user engagement with the intervention is posited as a requisite for effectiveness. On the other hand, engaging with the very technology used to deliver these interventions might be or feel detrimental to users’ well-being. In 2020, Erasmus University Rotterdam embarked on the development of a DBCI as a part of a comprehensive student well-being programme. Early user research conducted to design this DBCI presented this exact challenge. Some users acknowledged the potential benefits of the tool, but many had the perception that their smartphones were a source of stress.^11^ This tension between the need for user engagement and the potential stress associated with engaging with mobile technology raised a fundamental design challenge and created the contextual conditions prompting the pursuit of this research: how to create a DBCI that captures user engagement without relying on sustained usage.

### Usage and engagement

Designing an engaging tool that does not require sustained usage might initially sound like an oxymoron. Commonly, engagement with digital interventions has been equated to usage quantity or frequency.^4^ However, theories of engagement in digital behaviour change and Human–Computer Interaction (HCI) domains alike largely agree that quantity of usage alone does not equal engagement. Engagement is a multidimensional construct that must be understood not only through usage frequency, but also in broader behavioural, cognitive, and emotional dimensions.^4,5,7,12^ For example, a user integrating technology into their routine, deriving enjoyment from using it and understanding how to use it to reach their goals, also indicates engagement. As a result, design strategies that stem from a multidimensional conceptualization of engagement are geared towards optimising ease of use, enjoyment, and motivation as factors to encourage sustained usage.^7^

Some models extend the definition of engagement even further. Within the digital behaviour change field, engagement with DBCIs has also been conceptualised as a dynamic process that changes across time and includes two levels of engagement: micro- and macro-engagement. Micro-engagement encompasses moment-to-moment engagement with the digital intervention per se (e.g. discrete and temporary interactions with a mobile app delivering guided meditation). Macro-engagement encompasses engagement with the actual behaviour change process.^12^ The model does not explicitly outline what engagement with behaviour change entails but it generally extends the definition of engagement to include the individual's personal goals and intentions beyond the use of a DBCI. Notably this model proposes that in many cases, ceasing the use of a DBCI can be a positive sign, indicating that the user is independent and does not need any more support for maintaining their desired behaviours. Similarly, O’Brien et al.^13^ explore engagement with other types of digital products (not only DBCIs), and outline how dis-engagement is usually regarded as a negative outcome to be avoided, and argue that this is not necessarily true. Their position paper challenges the assumptions that engagement needs to be continuous and intense to generate positive results for a user. They also include the concept of agency into their model, framing feelings of agency as crucial to a desirable type of engagement. That is, the user being in control of the depth and frequency of usage of a system is crucial to them for engaging with a system in a way that is healthy and constructive. They conclude by posing disengagement as a natural part of the process of engagement and recommend considering high user agency when designing for engagement.^13^ This idea is also explored outside of the digital sphere; in a multidisciplinary overview of engagement, Bijkerk et al.^14^ drew a parallel between engagement with face-to-face interventions and engagement with DBCIs, and concluded that this notion can be extended to face-to-face interventions, where engagement might also happen away from the intervention space (i.e. a psycotherapy session).

### The current study

The notion of macro-engagement (or the exploration of the attributes of engagement and dis-engagement) outlines an opportunity. If engagement that happens outside of the intervention space can be influenced by design, it means there could be a possible avenue to solve the challenge of achieving positive results without relying on or encouraging sustained usage of a tool. Thus, the main goal of this study was to further understand macro-engagement and how to design for it in the context of DBCIs for well-being. To do so, we aimed to answer two main sub-questions: (1) what does macro-engagement look like in the context of DBCIs for well-being? And (2) how might we use design to encourage macro-engagement?

To answer these questions, we conducted thirty semi-structured interviews with three participant groups: mental health professionals working with clients; digital health experts, and users of well-being DBCIs. The groups were chosen because they provide a unique perspective about a part of engagement with DBCIs. Users can provide first-person accounts of engaging with DBCIs and behaviour change goals. Experts can add the perspective of designing and measuring engagement in DBCIs. Finally, we draw a parallel between face-to-face therapy and DBCIs as many DBCIs use direct translations of therapeutic practices within the digital context. Professionals delivering psychotherapeutic interventions can provide insights into engagement with the contents of many DBCIs, their mechanisms of action, and the process of change that clients undergo while following an intervention. Consequently, we analysed the qualitative data following a grounded theory approach.

Even though the concept of macro-engagement is increasingly acknowledged, studies that explicitly investigate it are still rare.^15,16^ Additionally, many design recommendations for engagement with DBCIs focus largely on momentary engagement with the technology by ensuring a positive or motivating experience for the user.^6,7,17,18^ To the best of our knowledge, this is the first paper that qualitatively investigates macro-engagement explicitly and outlines how this process looks offline, to draw implications for the design of DBCIs that effectively support behaviour change without encouraging sustained usage of digital technology.

## Method

### Study design

We conducted our study informed by the grounded theory method outlined by Charmaz.^19^ This methodology relies on a constructivist paradigm, viewing reality as subjective and posing it can be understood through individuals’ experiences and perspectives. It views the role of the researcher as a co-constructor of meaning, and thus encourages the researcher to acknowledge and consider their biases during the process. It also suggests iterative rounds of data collection and analysis, where researchers collect new data based on findings, and continuously refine theoretical concepts as the study progresses and provides a specific coding framework. This was deemed the most appropriate method since the research subject is directly related to subjective experiences and has been scantly investigated. We closely adhered to this method, with a strong emphasis on practices related to reflexivity and data analysis. Our application of theoretical sampling was somewhat constrained due to scheduling challenges and time constraints.

### Procedure

Data were collected through thirty semi-structured interviews conducted online through Zoom between June and December 2021. The recruitment of mental health professionals and DBCI experts happened in tandem and the recruitment and questions asked to the DBCI user group were influenced by initial insights that emerged from interviews with the first two groups. The first author conducted 25 interviews in English and two student assistants helped by conducting five of the interviews in Dutch and translating them into English. Interviews lasted between 30 to 60 min; they were video recorded, transcribed verbatim automatically by Zoom and subsequently manually revised against the recording to correct mistakes. Recordings were deleted and transcripts were anonymised once the transcription process was finalised to ensure the privacy of the interviewees. Consequently, the transcripts were entered into Atlas.ti for the analyses. Users received 10€ in remuneration for their participation; in turn, therapists and digital health experts received 50€, to make up for the potential time taken off work and mirror their hourly rates. Ethics approval for the study was given by the Ethics review Committee of Department of Psychology, Education and Child Studies (record no. 20-066a).

### Participants

Thirty individuals who belonged to three different groups of participants were recruited: mental health (MH) professionals, digital health experts and users of DBCIs. Participants were contacted with a description of the study and were asked for written informed consent before the interviews. Participants in each group were recruited until saturation of the data (i.e. when each new interview produced little novel data) was accomplished within each group.

#### Mental health professionals

While interacting with a digital tool is not identical to receiving in-person therapy, the interventions included in DBCIs for well-being and mental health are often digital translations of techniques and exercises used in psychotherapy. Therefore, at least one part of these processes is analogous; users are learning and practising how to deal with their thoughts and emotions through specific techniques. MH professionals were recruited through LinkedIn posts and emails sent through the authors’ networks. Participants who met the following criteria were included in the study: clinical psychologists or psychotherapists practising in Europe, with experience using Cognitive Behavioural Therapy (CBT) or Acceptance and Commitment Therapy (ACT), currently working with clients. Ten MH professionals were interviewed, seven identified as women and three as men. All of them used either CBT, ACT, or both in their practice. The mean years of clinical experience were 7.7 (range = 2–20; SD = 6.07).

#### Digital health experts

Professionals working primarily with digital health tools were also deemed relevant because of their experience in designing and testing these types of technologies. This group was recruited through LinkedIn posts and emails sent through the authors’ networks. Participants who met the following criteria were included: located in Europe and involved in researching, designing, and/or evaluating DBCIs and digital health services. Nine experts were interviewed. Six identified as women and three as men. Eight were academics, involved in university or research projects, and one of them was a manager in a company that offers blended mental health care. The mean years of experience in the field were 7.1 (range = 2–15; SD = 4.1).

#### Users of DBCIs

This group is particularly relevant to this study as they can directly narrate their personal experiences using digital tools for their well-being and behaviour change goals. This group was recruited through the first author's network and a database of students from Erasmus University Rotterdam interested in well-being. Participants who met the following criteria were included: individuals who were either habitual users, casual users, or users who had used a DBCI habitually before but had stopped using it. Eleven users of DBCIs for well-being were interviewed in this study, six identified as women and five as men. The mean age of these participants was 24.9 (range = 19–34; SD = 5.04). The types of DBCIs they used varied and some of the participants used more than one tool. Specifically, one participant used a running app, one used a migraine app, two used smartwatches and ten of them used a mindfulness app.

### Materials – qualitative interview script

Each participant group had different interviews, but all interviews were aimed at discussing (1) if and how macro-engagement might happen and (2) how can it be encouraged, from the perspective of each group. Therapists discussed topics like how they conceptualise and encourage patient engagement during and outside therapy sessions, strategies to encourage patient engagement, and the process of change in the therapeutic setting. Digital health experts directly addressed how they conceptualise micro- and macro-engagement in their projects by discussing the Yardley et al.,^12^ diagram of macro-engagement and how they think it could be encouraged. Finally, DBCI users discussed how they use digital tools to get closer to their goals, how tools influence their lives, how and when they become independent from them, and which challenges and strategies exist in that process. Table 1 depicts sample questions and interview flow for each group.

**Table 1.** Overview of sample questions used in interview guide per group.

Group	Sample questions
1.Mental health professionals	1.1 Tell me about yourself and your work 1.2 What do you think about when I mention the term “patient engagement''? 1.3 How do you determine when someone is ready to stop being in therapy? 1.4 Do you try to encourage patient engagement somehow? If so, how? If not, why?
2. Digital health experts	2.1 Tell me about yourself and your work 2.1 How do you define “user engagement’ in your work? 2.3 How do you measure engagement in your projects/team/practice? 2.4 [Show engagement model proposed by Yardley et al.] What do you think…? 2.5 Do you incorporate this engagement outside the intervention in your projects at all? If so, how?
3. DBCI users	3.1 Tell me how you use your tool and what for 3.2 Why do you continue to use it/Did you stop using it? 3.3 What benefits/outcomes do you notice from engaging with your tool? 3.4 What do you think encouraged or facilitated these outcomes or changes? 3.5 Can you think of actions, circumstances or situations outside of the use of the tool that have helped you achieve this change/outcome? If so, how?

### Data analysis

Data were analysed separately for each group following the grounded theory approach methodology outlined by Charmaz.^19^

As per Charmaz's^19^ procedure for data analysis, the first author, a PhD candidate in behavioural sciences with a background in digital design, analysed 100% of the transcripts following these steps: (1) initial coding, where each segment of data got assigned conceptual codes, (2) focused coding, where the initial codes are evaluated and the most relevant codes are used to categorise the data more concretely, (3) axial coding, where codes are grouped into thematic clusters and subcategories, and (4) thematic coding, where the resulting themes are conceptually connected into models of how the three groups experience engagement from their perspectives. To assure reflexivity in the process, the main researcher kept a researcher journal. Additionally, a secondary coder, who was interning on the project and a health psychology and behaviour change master student, analysed 50% of the transcripts per group. The secondary coder followed steps (1) (2) (3) and (4). Before the start of the coding, the hierarchical relationship between the coders (supervisor–student) was addressed and an agreement was made that neither perspective would be favoured over the other. While the master's students had less expertise in digital interventions, their background in (health) psychology brought richness to the discussions. Discussions between the coders were held at each step of the analysis to visualise overlaps, question assumptions and possible biases, and to resolve conflicts in codes. Where conflicting codes appeared, coders had conversations to support their reasoning, wherever agreements were not reached, this was noted and mentioned in the discussion section. The first author integrated the final codes and themes first for each group and then compared them across groups, triangulating the results into a theoretical model.

## Results

The themes and codes generated from the interviews were broad as interviews included various aspects of the experience of supported behaviour change. Hence, only themes that relate to the main research questions will be explored in detail in this result section. A complete overview of codes and themes is presented in the code trees in Appendices A1.1 to A1.3.

### Mental health professionals

The therapeutic process was discussed in general, with special emphasis on patient engagement. Most MH professionals described engagement as that happening not only during sessions but also beyond the sessions, except for one, who said they did not conceptually see the split between the process in the session and outside. Notable themes emerged regarding engagement beyond the session, including how it manifests outside of the therapeutic setting and how to encourage it. Table 2 includes the overview of these themes and related sub-themes, whereas each sub-theme is described in more detail below.

**Table 2.** MH professional group themes, sub-themes and codes related to macro-engagement.

Theme	Subtheme	Codes	Quotes
1. Engagement beyond the session	1.1 Varied efforts exerted outside of sessions	Complementing practice with other initiatives Complying with homework Thinking about therapy outside therapy Working with others/ including others	‘So when someone starts sharing what he is doing or calls on the support of others. I usually think that is a sign that someone is in good therapy mode.’ (MH professional 6)
2. Positive outcomes of process	2.1 Non-reliance (aka patient becomes own therapist)	Confidence/ initiative Learning and implementing skills	‘I think when you feel like someone is becoming or became their own therapist, so someone is very good at observing what is happening and reflecting on themselves and making comments like oh I’m doing this again… and someone is feeling content about doing so… I think that's a sign’ (MH professional 1)
3. Strategies to encourage patient engagement	3.1 Strategies to encourage efforts beyond the session	Broadening the scope Explicit about the importance of independent work	‘We always talk about what they have done [in therapy], and then I try to make a connection with daily life, so they can see that that's useful, and I can relate to that, or I can use that in my daily life.’ (MH professional 10)

#### Subtheme 1.1. Varied efforts exerted outside of sessions

When discussing patient engagement that happens beyond the therapy sessions, MH professionals discussed signs which indicated that the patient exerted effort outside of the session to implement the learnings achieved therein, during their normal life. This was in some cases deemed more valuable to the process than engagement during the session itself. Therapists gave varied examples of efforts beyond the session. Nine mentioned complying with homework (i.e. commitment), four mentioned involving loved ones in their process (i.e. seeking social support), four mentioned patients complementing their process with other initiatives, like books or classes (i.e. commitment), and two mentioned thinking about what was discussed during the sessions (i.e. reflection).

#### Sub-theme 2.1. Non-reliance or independence

Nine of the MH professionals outlined ways in which they could see that their clients gained new abilities that made them less reliant on support. Specifically, this meant the client gaining the confidence or initiative to solve their own problems, and learning and implementing therapeutic strategies. This sub-theme includes mentions of positive outcomes and parts of the process of engaging with the process without support. These codes emerged from a conversation about outcomes of therapy, but it was considered to have a connection to the concept of macro engagement as it is more of a proximal outcome that manifests outside of the session and part of the process towards the patient feeling better.

#### Sub-theme 3.1. Strategies to encourage engagement beyond sessions

MH professionals use a wide variety of strategies to keep clients motivated and committed to their therapeutic process. Seven of them mentioned strategies specific to trying to encourage clients to do work independently in between sessions. Specifically, three therapists mentioned trying to broaden the scope of the session by suggesting the client to think about what was discussed in therapy during their daily life or by giving them assignments. Five of them described the importance of the client understanding that change requires not only coming to therapy, but also constant work. They described achieving this, by explicitly discussing with the client the importance of work between sessions.

In summary, for most MH professionals, engagement beyond the session (macro-engagement) could be seen as just as important as engagement during the session. In their view, this manifests as different efforts exerted by the client in the time between sessions and by a demonstration of non-reliance. They encourage this engagement beyond sessions by explicitly encouraging the client to do homework or think about therapy between sessions.

### Digital health experts

This group discussed their own experiences and definitions of engagement with DBCIs. All experts recognised and entertained the idea of macro-engagement and notable patterns emerged regarding how macro-engagement might manifest, how to encourage it and how to measure it. Table 3 includes an overview of the main themes and related sub-themes. Each sub-theme is explored in more detail below.

**Table 3.** DBCI expert group themes, sub-themes and codes related to macro engagement.

Theme	Subtheme	Codes	Quotes
1. Engagement beyond a DBCI tool	1.1 How to identify engagement beyond a DBCI	Learning and learning transfer Showing self-efficacy Understanding personal goals and putting efforts towards them Performing desired behaviour	‘I'm also thinking about the transfer of the skills or the transfer of the knowledge and applying it in your own situation’ (Expert 2)
	1.2 Strategies for engagement beyond a DBCI	Explicitly informing user Using objects and props Accountability and peer contact	‘…for instance, people who shouldn't drink alcohol, have like their own personal bracelet and this bracelet is what reminds them [of their intentions] and then that thing that they take everywhere…it's not technology’ (Expert 5)
2. Considerations about measurement and evaluation	2.2 Challenges in measurement and evaluation	Depend on context, target behaviour, and personal differences Macro and micro engagement are difficult to distinguish Unreliable measures or constructs	‘The tricky thing is indeed…what are you measuring then, is it really engagement… or is it more of like a predictor or outcome of engagement…that's tricky I don't know… I'm also not sure if there are a lot of questionnaires on engagement with behaviour in itself.’ (Expert 7)

#### Subtheme 1.1. How to identify engagement beyond a DBCI

When asked about the concept of macro-engagement, eight experts discussed aspects that could be used to identify engagement beyond a DBCI. Specifically, four experts mentioned that the users knowing what their goals are and understanding how the DBCI helps them could entail macro-engagement. Four of them mentioned that this could include learning and transferring skills from the DBCI to their daily lives. Five of them viewed it as users displaying self-efficacy and five mentioned that achieving the target behaviour of the DBCI could also be catalogued as macro-engagement.

#### Subtheme 1.2. Strategies for engagement beyond a DBCI

Six experts discussed either how they had tried to influence engagement beyond a DBCI in their projects or how they would go about it in theory. Three of them mentioned that a way to influence this is explicitly informing the user, meaning the DBCIs could include information to let the user know they should practise by themselves, linking them to other resources or letting them know the tool will not always be there to support them. Another set of strategies mentioned by three of the participants was to involve others in the process (i.e. social support), suggesting that including people for accountability or any sort of peer-to-peer contact was a good way to encourage this. Three experts mentioned that using objects and props (i.e. cue associations) could be a way to foster engagement beyond the tool.

#### Subtheme 2.1. Challenges in measurement and evaluation

Five of them noted how micro-engagement and macro-engagement would depend on the context, target behaviour and personal differences of the user. Four of them discussed how measuring macro-engagement would be difficult because of unreliable or undefined constructs. Two of them signalled that micro-engagement and macro-engagement were difficult to distinguish. Notably, one of the experts, who had explicitly studied engagement, mentioned that the concept of macro-engagement was interesting, but the distinction between micro- and macro-engagement not only was difficult but it might also not be necessary.

In summary, experts could somewhat identify macro-engagement in their projects and experiences. In their perspective, it manifests through the user understanding how to use the tool to reach their goals, learning transfer, performing the target behaviour and displaying self-efficacy. They encourage this engagement beyond sessions by explicitly including information and additional resources, using props or including the support of others. Most conversations delved into how to measure macro-engagement and underlined how difficult this would be.

### DBCI users

Users discussed their own experiences using DBCIs and how they impacted their lives. Most of them discussed the behaviours they performed without the explicit support of the digital tool, whereas three of them did not seem to be able to think about engagement or actions offscreen. These users were specifically: one of the mindfulness app users, one smartwatch user, and a participant who used a running app and a mindfulness app. In the users’ interviews, two sub-themes emerged: an account of the different efforts they placed outside of the use of technology to reach their goals and moments where they noticed that they could activate knowledge and skills without support (see Table 4). The sub-themes specific to engagement beyond the DBCIs are outlined in more detail below.

**Table 4.** DBCI user group themes, sub-themes and codes.

Theme	Subtheme	Codes	
1. Engagement beyond the DBCI	1.1 Efforts placed outside the use of technology	Giving themselves accountability Intentionally finding other contexts and moments to practise skills Making extra efforts (misc) Putting effort to make the behaviour a habit Self cues and reminders	‘[The app] sort of asks you to be present during your day. So every time I remembered to do it, I drew a mark on my arm, to kind of make it conscious that I was doing it and to remind me to keep doing it. Later I just got a tattoo of a pause sign on my arm to remind me to stick to it…’ (User 1)
	1.2 Acquired skills and abilities	Being able to regulate emotions and stress Being mindful/aware Being able to focus Not needing the tool for support New knowledge	‘Yeah, I was able to meditate wherever, whenever and I didn't feel like I needed to be like I needed the app to do it…so I was free, and I knew the secrets of it or how to do it.’ (User 2)

#### Subtheme 1.1. Efforts placed outside the technology

Eight participants discussed strategies or moments where they put extra effort outside of using the DBCI to make sure they reached their goals. All eight mentioned finding other spaces or contexts to practise the behaviour on their own, for instance, going on mindful walks or using the commute as a moment to practise mindful awareness without using the DBCI. Three users said they gave themselves cues and reminders to trigger their desired behaviour. For instance, one mindfulness app user got a tattoo to make sure that on every time they seeing it, they remember to bring their awareness to the present. Five participants consciously tried to make their behaviours a habit by embedding them in their routines, for example, by having specific moments of the day when they would perform their behaviours. Four users tried to give themselves accountability by, for instance, involving loved ones in their process and reminding each other of their goals by sending each other reminders or attempting to do things together. Seven people narrated other instances of putting extra effort into their goals like using multiple tools and resources, such as a habit tracker to remember to meditate or complementing their mindfulness practice with journaling.

#### Subtheme 1.2. Acquired abilities

The same eight participants were also able to narrate moments in which they recognised they acquired new abilities and implemented them during their daily lives. Three mentioned the ability to focus better, six outlined gaining new knowledge that impacts how they behave, five said they were more mindful or aware of themselves, six discussed being able to regulate emotions and stress better, and finally, seven of them discussed explicit moments in which they could apply these skills without the support of the DBCI.

In summary, while not all DBCI users were able to point out instances of macro-engagement, the ones who did outlined efforts placed offscreen and moments where they could activate new skills away from their tools.

### Qualitative comparison of themes across the groups

The three groups outlined a wide array of events that happened outside the realm of support and in looking at those instances, a pattern emerged. These instances could be catalogued into two categories.

The first category includes activities that complement the undergoing behaviour change process. In the expert group, this emerged as a smaller code: “understanding their goals and putting efforts towards them”. In the MH professional group and DBCI user groups these instances emerged as a subtheme, “efforts outside of the session” and “efforts placed outside the technology”, respectively. The second category includes instances that can be catalogued as positive results of engaging with a DBCI. In the experts’ group, this emerged as codes and it included: “transfer of learning”, “showing self-efficacy” and “performing the target behaviour”. In the MH professional and DBCI user group, it emerged as a sub-theme “non-reliance (aka patient becomes its own therapist)” and “acquired skills and abilities”, respectively.

By contrasting these two categories, we see that there is a common factor of intent. The first category requires deliberation: extra efforts made consciously to adhere to goals and target behaviours in daily life and away from a DBCI. The second one requires less deliberation and thus happens more spontaneously: skills and knowledge gained through a DBCI are applied in daily life, or target behaviour is achieved without much deliberation or planning.

While the three groups shared commonalities, some differences emerged that should also be considered. First, contradictions emerged in outlining the existence of macro-engagement. Macro-engagement was homogeneously discussed as important in the MH professional group while in the expert group, it was mainly discussed in theory, and not all DBCI users were able to discuss it. Second, differences emerged in the relevance given to macro-engagement. In the MH professional group, it was highlighted almost as important as engagement during the sessions; in the expert group, one expert questioned its relevance; in the user group, users who relied solely on the DBCI could not see the importance of engaging with their behaviours in other ways (i.e. their goal was intimately linked to the usage of the tool). Finally, differences appeared in discussions that were about delimiting where micro-engagement ends and macro-engagement begins. In the expert group, it ranged from very proximal instances, such as understanding how a DBCI helps personal goals, to more distal outcomes like performing the target behaviours in real life. In the MH professional group, however, this shift was viewed as linked to more proximal process outcomes related to the therapy progress, like putting effort into the process and learning transfer, but not including distal therapeutic outcomes (i.e. improvements in relevant indicators of wellbeing).

### Reflection on the coding process

During the coding process, the two coders were able to reach agreements on the majority of codes, themes, and their structure; however, the distinction between macro-engagement and micro-engagement remained a point of ongoing debate. The primary source of contention was whether certain behaviours or attitudes should be classified as macro- or micro-engagement. In the case of the MH professional group, these events included mentally preparing for the session and doing homework, while in the DBCI user group, it involved the user's efforts to integrate the tool into their routine.

Furthermore, the definitions of macro-engagement slightly varied across the groups, which required the coders to examine and reconcile the differing perceptions of macro-engagement. CV, the main researcher, influenced by her background in digital design, viewed this process through the lens of the tool. Hence, the distinction between micro- and macro-engagement seemed very separate as they are two different spaces of interaction; onscreen, and offscreen. On the contrary, AT, the second coder, influenced by her background in health psychology viewed this process through the lens of the individual. In this light, the distinction between the two spaces seemed less relevant and codes were viewed more as indicators in different stages of a behaviour change process.

Recognising that the engagement process with DBCIs involves an interaction between a tool and an individual, the two perspectives were essential for questioning assumptions, developing a theory, and defining the boundaries of this process. Therefore, in the next section, a theory and its limits are drawn up.

## Discussion

Using a grounded theory approach, this study explored two main questions: first, what macro-engagement looks like in processes of behaviour change using support (digital tools or psychotherapy); second, how to design to facilitate macro-engagement. To make this section more succinct, a parallel is drawn between the therapeutic session and a digital tool. From now on, for simplicity, when discussing ‘the tool’, this encompasses both therapy sessions and DBCIs.

### What does macro-engagement look like in the context of DBCIs for well-being?

The first aim of this study was to understand how macro-engagement looks like in behaviour change processes supported by DBCIs. By triangulating the perspectives of the three groups, we could outline that macro-engagement might manifest through a wide array of offscreen behaviours, attitudes and thoughts. These results support Yardley et al.'s theory of micro- and macro-engagement^12^ and O’Brien et al.'s reframing of dis-engagement.^13^ It shows how the introduction of a DBCI creates two spaces: the space where the user interacts with the tool, and the space where the user might work towards their goals or target behaviour unsupported. Moreover, the accounts gathered through these semi-structured interviews provide an additional layer of information, characterising how macro-engagement might be influenced by the intent required from the individual, which can go from deliberate to spontaneous. This resonates notably with Zhang et al.’s^20^ adaptive decision-making framework, which states that in a DBCI-mediated behaviour change process there is an action level (i.e. off-screen in everyday life) made up of small decisions. In this action level, this model describes how the daily decisions a person makes (concerning their goals) can be influenced either by automatic decision making (habits) or by a deliberate attempt to adhere to goals (i.e. goal-directed behaviour). This finding is important because it outlines an opportunity to support engagement beyond the screen: making deliberate efforts less effortful by design.

**Figure 1.**
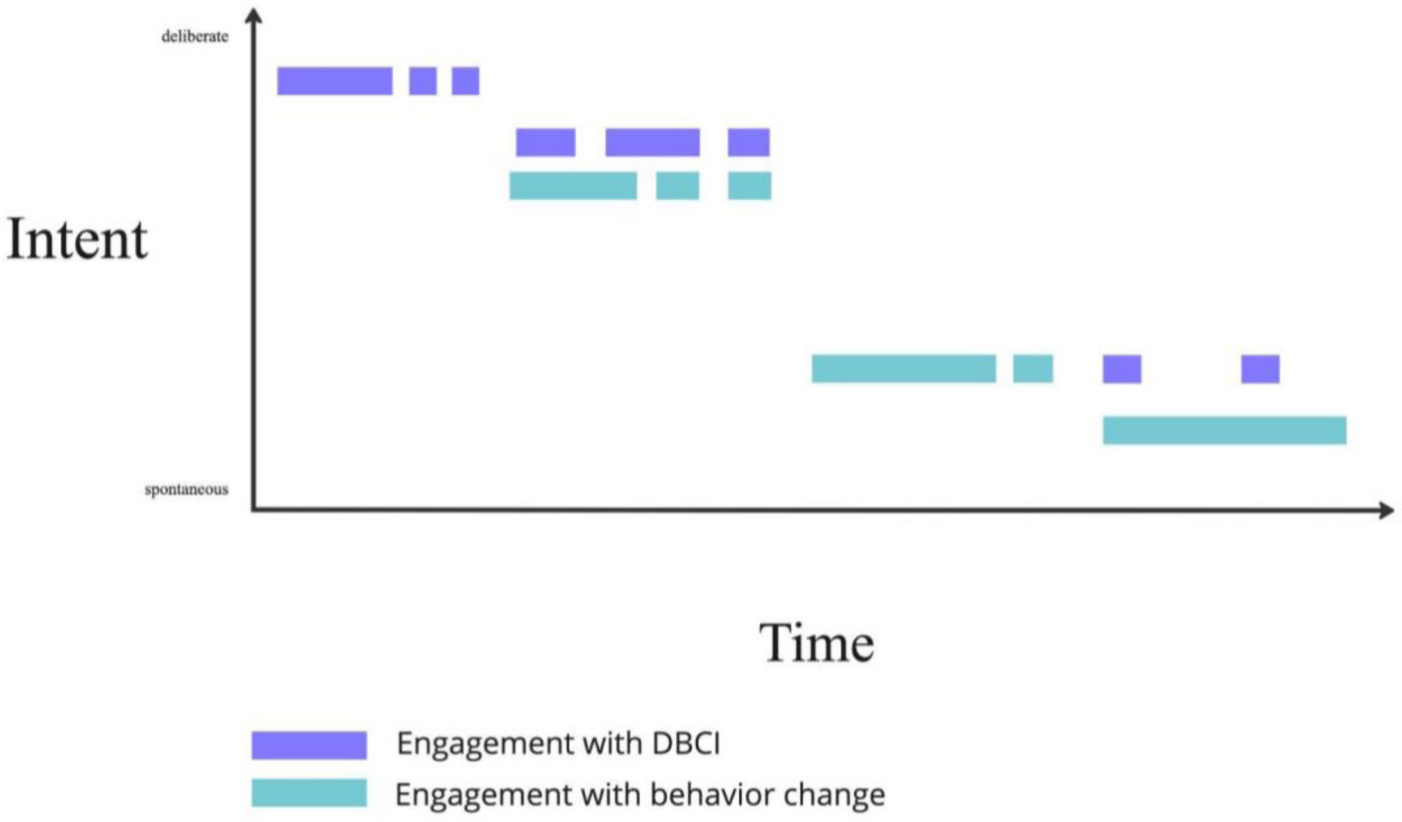
Engagement with DBCI and beyond the DBCI over time and with the intent required.

Figure 1 is a hypothetical example of how a behaviour change process mediated by DBCI might happen. It is inspired by Yardley et al.'s^12^ illustration of micro- and macro-engagement, but includes and focuses on a new aspect: intent required. It outlines how onscreen and offscreen engagement varies over time, might happen in tandem and require different levels of intent as time goes by. In this hypothetical example, the user started by engaging only with a DBCI and then complemented it with deliberate efforts offline, moving on to not needing the support of the digital tool and having to apply less and less intent to adhere to their target behaviour as it becomes integrated into their functioning.

### Limitations of the model

Contradictions emerged when triangulating the three groups and difficulties arose in the data analysis process when the two coders were co-constructing meaning. Based on these contradictions and challenges, we outline the caveats of this model. First, macro-engagement might not always take place. Second, it might not always be important to a behaviour change process. Third, macro-engagement might be too broad and difficult to operationalise.

#### Macro-engagement might not always take place

While the MH professional and digital health expert groups could homogeneously identify how macro-engagement could look like, the DBCI user group offered varied perspectives. In this group, three users could not identify any behaviours performed without the support of their tool to reach their goals. These users had some characteristics in common. First, two of them were users of tracking tools, namely, a running app and smartwatches; thus, they shared a usage intention: monitoring behaviours. Two of them also used mindfulness apps but their usage of the app was palliative; they did not use it to train mindfulness skills often, but they used the tool to feel better if they had a specific challenge. So again, the intention (i.e. de-stressing) was shared between these two users. This suggests that offline, unsupported efforts may not always be present in a DBCI-supported behaviour change process. The reason why some processes may not include offline, unsupported efforts is still unclear. The results could suggest that this can be related to the intentions of the user and the complexity of the behaviour to achieve. Based on the findings, it could be inferred that some types of goals have higher learning curves or require different efforts, for example, becoming mindful vs. running more often or better. Becoming mindful involves learning about attention, adhering to a meditation practice often, finding moments in the day to exercise mindfulness and remembering to apply these newly found skills on the go in daily life before this becomes effortless behaviour. Whereas using an app to track runs requires a commitment to an activity being embedded repeatedly into one's routine, but not particularly learning a new skill. Another possible explanation for these results is that the group of users that could point to offline, unsupported efforts – all users of mindfulness apps – have become more mindful of their experience, and hence their pervasive efforts. However, there is not enough data to assert these theories. More research would be needed to further understand the offline behaviours of users of different types of tools.

#### Macro-engagement might not always be relevant

Through the discussion of macro-engagement, its relevance was also discussed both implicitly and explicitly. Discussions in the MH professional group often touched upon the importance of independent efforts. For them, sessions are a training ground, but the work needs to be done every day individually. In short, macro-engagement is a very important and desired part of the process in face-to-face therapeutic settings. Conversely, the results are more heterogeneous in the DBCI user and expert groups. In these groups, contradicting patterns emerged that questioned the importance of macro-engagement in a process of behaviour change aided by a DBCI. For instance, users of trackers and monitoring apps always performed their behaviours supported by their respective tools and did not consider not using the tool. On the other hand, two of the users of meditation apps felt like they did not need the tool anymore, they had learned something that they could implement in daily life, and this was viewed as a sign that they achieved their goals. In the expert group, there were mixed perspectives. For example, one expert working on a smoking cessation app discussed that they considered engagement beyond the usage of the system in their project from the start. In contrast, one of the experts questioned if the distinction between micro- and macro-engagement was even that relevant, mentioning how most people would not differentiate between what they do with a tool and what they do outside of the tool.

Based on these findings, one could infer that first, whether macro-engagement is relevant and takes place in a DBCI-supported behaviour change process depending on the goal of the user. This mirrors another prominent model of engagement: the perfect interaction model, which posits that the *intentions* of the users drive how they engage with a DBCI.^21^ For example, from the perspective of a user of a tracking tool, independence seems irrelevant; if they are using a tool to track their exercise and they are exercising more because of it, then, why would it matter if they exert efforts without the tool or not? On the other hand, looking at the overlaps between the group of MH professionals, the users of mindfulness apps and the smoking cessation app expert, it can be inferred that non-reliance on a tool is at least sometimes a part of the process and an aspect of the engagement. These overlaps happened when the target behaviours needed to be learned and rehearsed, and would eventually need to be applied spontaneously in daily life. For example, being able to identify difficult emotions and respond appropriately, or curbing smoking cravings on the go. Furthermore, this suggests that when digital tools are intended for learning and training skills, being able to apply those skills independently (learning transfer) can be a desirable type of macro-engagement.

#### Macro-engagement is difficult to operationalise

To be able to design for macro-engagement and evaluate the impact of design strategies and choices, it would be important to be able to operationalise the construct. However, through discussions with the digital health experts and through reflecting on the analysis process, it was found that operationalizing macro-engagement for DBCIs is challenging in two ways: identifying where macro engagement begins and ends and identifying concrete indicators that apply to all DBCIs for well-being.

First, identifying where one space ends and the other one begins proved contentious during the analysis phase and thorough discussions were needed to reach a consensus. The categorization of instances like mentally preparing for a therapeutic session and doing homework in therapy, or putting effort to make the use of the tool habitual, was challenging. These events were difficult to categorise in coding because they exist in a liminal space; they are not performed fully independently or do not happen completely outside of the space of support. This was also concretely mentioned by the digital health expert group, saying that drawing those boundaries between micro- and macro-engagement would be challenging.

Second, identifying concrete indicators that applied to all DBCIs proved difficult as all groups mentioned a wide array of behaviours, thoughts and attitudes of different scopes. This was possibly due to the variety in the types of behaviours and digital tools included in the study. A reflection of this is evident in the literature too. When macro-engagement is evaluated, there is some agreement that it entails some sort of offline interaction with the behaviour change goals, but there is no apparent agreement on what that specifically involves. Some studies equate macro-engagement to performing the target behaviour outside of the tool, that is to the achievement of the target outcome of the intervention. For instance, in a study conducted by Fodor and Balázs,^22^ it was operationalised as participating in a real-life activity as a part of a virtual intervention. Molloy and Anderson^15^ did a review of digital interventions for depression where macro-engagement was also operationalised as performing target behaviours in daily life. In contrast, some studies deal more with learning and how that is transferred to other contexts. For example, Lally et al.^16^ evaluated macro-engagement in a stress self-management program through a self-report of how much participants used what they learned in the digital program during their daily lives. This suggests that macro-engagement is more of an umbrella term encompassing behaviours, attitudes and thoughts that happen away from the tool and its operationalization is dependent on the intervention targets and working mechanisms.

These findings hint towards two ideas. First, a more viable way to think about micro- and macro-engagement is as a continuum of engagement, instead of separate categories in which an individual can be engaged at different distances from the DBCI: from exclusively being supported by the DBCI to not using the DBCI at all but being engaged with its contents offscreen. Second, “DBCI for well-being” is too broad a category to be able to operationalise macro-engagement. This finding is congruent with Yardley et al.'s^12^ recommendation to operationalise macro-engagement in *context*. It also confirms findings from a systematic narrative review of engagement conducted by Kelders et al.,^4^ in which they suggested that the wide variety of digital health technologies might require definitions of engagement specific to a type of technology and its context of use instead of an overarching definition of engagement for all digital health technologies.

### How can we use design to encourage macro-engagement?

The second aim of this study was to determine how to design to encourage macro-engagement. Through the discussions with participants, we were able to identify which actions were taken to encourage engagement beyond the tool. The MH professionals encouraged this by explicitly discussing the importance of the clients' independent efforts and by broadening the scope, telling the clients to try strategies out alone and giving them assignments. Digital health experts mentioned adding information for the user to expect eventual non-reliance and involving peer-to-peer contact, props or objects to nudge the user to practise skills and remember goals during daily life. The DBCI users engaged beyond the tool by finding other contexts to practise their skills, giving themselves reminders and accountability, and trying to develop strong habits. These results yielded two main ideas for encouraging macro-engagement in skill training processes. First, the role of the users' environment should be considered. Second, the transfer of the learning process appears to be an adequate design rationale for engagement beyond the screen. Each idea is explored below in more detail.

#### The role of the environment should be considered

Macro-engagement,^12^ or dis-engagement,^13^ theories state that non-usage is richer than it appears; not engaging with tools does not mean not engaging with goals and target behaviours. The findings of this empirical, qualitative study support this notion and outline how a lot of the engagement beyond the tool relies on interactions between the users and their environment offscreen.

Thus, a way to encourage these pervasive user efforts would be to extend the intervention and make the content of a DBCI available through the users' physical surroundings. A similar idea was explored by Peters et al.'s METUX model.^23^ In this model, authors propose the concept of spheres of influence that go beyond the interface of a system. In other words, they suggest that designers not only consider how a user interacts with a system interface but also how they engage with technology-enabled tasks and how this impacts overarching behaviour offscreen.

In view of these results and with the rise of increasingly “smart” devices, it might be tempting to add pervasive technology to the mix to create engaging DBCIs, that is extending interventions through smart home devices. However, embedding more technology and more content into the users' context could start contradicting the initial motivation for this study: users being sceptical of the amount of time they spend on screen. Simply making a DBCI part of their whole life could go against that. Thus, how this is done matters. Fortunately, the field of HCI has had an impetus towards considering and circumventing the possible negative effects of technology on users for a while.^24^ In their review of engagement in the HCI field, O’Brien et al.^13^ conclude on the importance of considering user agency, allowing the user to engage with digital technology knowingly and intentionally. This could entail encouraging goal-directed behaviours and designing technology that allows the users to choose to engage or not. Another source of inspiration could be Calm Technology. This approach posited that technology would become pervasive, and argued that in such a panorama it is important that not all interactions require undivided attention. Thus, Calm Technology concerns itself with embedding parts of the experience with technology in the users' physical surroundings.^25,26^ Peripheral nudges and interactions are at the centre stage of this approach. Peripheral nudges are cues and reminders that are unobtrusive, as they are part of the context. An example of a peripheral nudge is The Ambient Umbrella3, which has a handle that lights up when it is going to rain; a subtle, unobtrusive nudge to remind people to pack their umbrellas on a rainy day.^25^ Peripheral interactions go a bit further, aiming to reduce the mental load to an absolute minimum by allowing the user to perform the easiest, least burdensome action possible.^26^ For example, the Amsterdam Rijksmuseum contains, in its pocket watch collection, an antique clock with a system of different sounds that announce time without the need to look at the clock. Moreover, some widely marketable products make use of peripheral interactions: most wireless headphones allow users to change songs and control the volume through very easy hand gestures without the need to take out their phones. This body of work can be very useful, particularly in utilising the users’ context to help them remember and practise their skills in a way that considers their attention, time and effort.

#### Transfer of learning as a design rationale

The findings also outlined that when DBCI users are learning a new skill, offline efforts would be enacted to remember, practice, and activate new skills gained through the DBCI in a different space. All these characteristics overlap with the construct of *transfer of learning* (also mentioned explicitly by some experts during the interviews). Transfer of learning, broadly speaking, refers to the application of skills or knowledge from one context to another.^27^ Thus, designing affordances that facilitate the transfer of learning might be a productive endeavour to solve the initial challenge: a tool that does not rely on increased usage for positive results.

Transfer of learning (and transfer of gaming) is widely recognised in the “*serious games”* literature. Thus this is an apt place to start as this research area has outlined recommendations and recipes to guide a design process that focuses on transfer. One of the most pervasive recommendations is to determine what exactly needs to be transferred.^28,29^ Another common recommendation is to identify the application environment and make explicit connections to it.^28,29,30^ However, in a review, Kuipers et al.,^30^ found that transfer of learning is rarely addressed as a design rationale in DBCIs for health – it is more often described as an outcome. Similarly, other researchers have suggested study designs that explicitly outline the design rationale used to influence transfer and evaluate how these included elements influence the transfer of learning.^29,31^ This indicates that more research is required to determine how the transfer of learning unfolds in DBCIs, how to support it through design and what attributes need to be considered in doing so.

## Limitations

This study had some limitations. First, the secondary coder in the process was a student interning under the supervision of the first author. To mitigate any power imbalances in the coding process, the first author and second coder agreed on a set of expectations about the process where both perspectives would be considered as having equal weight. Even with this procedure in place, it is difficult to know if the roles of the student and supervisor had no influence on any of the conversations or results. Second, we partially followed the iterative data collection and analysis recommended in the grounded theory methodology, but we did not manage to adhere to it completely due to time constraints and scheduling challenges. With enough time and iterations, a more certain model might have been offered as a result of this research. For instance, the recruitment of mental health professionals exhibited shortcomings. While incorporating this was pivotal for outlining individuals’ macro-engagement with the content and techniques of mental health DBCIs, further recruitment would have involved mental health professionals or other professionals administering face-to-face interventions focusing on a wider scope of behaviours like healthy eating or physical activity. Unfortunately, logistical constraints impeded us from continuing the recruitment of more participants. Additionally, our sample resulted in 20 women and 10 men and an overwhelming majority of researchers working in academia in the expert group. Having a more balanced sample could have indicated if there were differences between gender identities or professional fields in the experiences regarding macro-engagement. Finally and perhaps more importantly, the experiences of all participants were being recalled in retrospect; this means that recall bias plays a role in the results and limits the scope of the findings. Further research on macro-engagement should be conducted through diary studies or methods that reduce recall bias to further understand how this process takes place.

## Conclusions and further directions

This study was born out of a need to solve a design challenge that some DBCIs might encounter: achieving positive results without relying on or encouraging sustained usage. The notion of macro-engagement (or dis-engagement) as a rich space, posited a possible sphere of influence for design. Hence, this study set out to identify what macro-engagement with DBCIs for well-being looks like and occurs, and based on that, to make design recommendations to support it.

 This study introduces a theory of macro-engagement describing what macro-engagement could entail and under which circumstances it might appear. The results suggest that when macro-engagement is relevant and takes place, it does so through offline efforts that can be catalogued as intentional or spontaneous. The results also highlight that users of DBCIs do not always exercise macro-engagement and that this is not always essential to their behaviour change goals. Additionally, this study suggests macro-engagement may be better understood as an umbrella term for interactions beyond the screen and thus, in need of contextualization in relation to the targets of a DBCI, mechanisms of change, setting and population. Hence, the theory developed is limited and might not be applicable to all DBCIs. Further research should focus on understanding which types of (digital) tools or target behaviours should consider macro-engagement from the start and find ways to operationalise the construct for design and evaluation.

Notably, this study also sheds light on design implications for DBCIs that are aimed at learning and skill training, where macro-engagement overlaps with learning transfer. Thus, when a learning process is taking place, it may be fruitful to design technology that explicitly encourages and supports learning transfer to allow the user to progressively become independent of the tool. Calm Technology, explicitly designed for learning transfer, and other design approaches that create connections between the technology and the user's physical space are proposed as possible avenues to design for macro-engagement. This opens an interesting avenue for further research to design and investigate DBCIs that employ elements outside of the screen to extend the intervention space and to include interactions that support the user's learning process offscreen in order to evaluate how these features affect the transfer of learning and experiences of its users.

## A. APPENDICES

### A1. Code Trees

**A1.1 Table 1.** MH professional group themes, subthemes and codes:

Theme	Subtheme	Codes
Engagement with the session	Patient ownership	Being able to disagree and thinking along Taking initiative, guiding the process Attending well Making space
	Comfort and ease during session	Opening up Presence Showing engagement with their bodies
Engagement beyond the session	Varied efforts exerted outside of sessions	Complementing practice with other initiatives Complying with homework Thinking about therapy outside therapy Working with others/ including others
Barriers and facilitators that might influence engagement	Patient traits/states	Mood and emotions Patient interest/motivation Patient passiveness Patient readiness Willingness to work/ put effort
	External circumstances	Having a good therapeutic relationship Life getting in the way Seeing benefits
Strategies to encourage patient engagement	To encourage therapeutic relationship	Empathetic feel Gaining the patient's trust
	To encourage motivation in patient	Discussing the process and its hurdles Goal setting and monitoring Openly discussing the “why” /motivations Being flexible and gentle
	To encourage efforts beyond the session	Broadening the scope Explicit About the importance of independence
	Recommendations for digital tool	Ease Rewarding Visualising and rewarding progress Personalizing/ tailoring Compassionate voice feel Feeling of support
Positive outcomes of process	Improvements and goals met	feeling better Reaching goals
	Non reliance (aka patient becomes own therapist)	Confidence/ initiative Learning and implementing skills
Nuances and considerations	In the process	Context influences outcomes Engagement doesn't always lead to outcome Strategies depend on therapeutic principle
	In identifying engagement	Indicators depend on personal differences Indicators depend on what needs to change.

**A1.2 Table 2.** Digital health expert group themes, subthemes and codes:

Theme	Subtheme	Codes
Engagement with DBCI tools	How to identify engagement with a tool	Tool is part of habits Usage and Adherence Cognitive and emotional dimensions
	Factors that contribute to engagement with the tool	Appealing, fun experience Considers phases of behaviour change Ease Non-judgemental support and warmth Fit and relevance of the tool Personalisation/tailoring Notifications Safety and trust Satisfaction and progress
Engagement beyond a DBCI tool	How to identify engagement beyond the tool	Learning and learning transfer showing self-efficacy Understanding their goals and putting efforts towards them Performing desired behaviour
	Strategies for engagement beyond the tool	Explicitly informing user Using objects and props Accountability and peer contact
Considerations about measurement and evaluation	Challenges in measurement and evaluation	Engagement accompanies a process of change, sometimes out of the scope of a study Depend on context, target behaviour, personal differences Macro and micro engagement are difficult to distinguish Unreliable measures or constructs
	Usage measures alone are too narrow	Qualitative research is needed to understand experiences better Usage data needs more context Adherence does not equal engagement
	Narrating previous experiences with measuring engagement	Engagement is important for effectiveness Having focused only on outcomes, not engagement
Nuances and considerations	In the process	Context influences outcomes Engagement doesn't always lead to outcome Strategies depend on therapeutic principle
	In identifying engagement	Indicators depend on personal differences Indicators depend on what needs to change.

**A1.3 Table 3.** User group themes, sub-themes and codes:

Theme	Subtheme	Codes
Motivation to start	Intentional	Managing existing problems
	Casual	Social influence Vague intention of self-improvement Curiosity
How tools are used	Usage patterns	Habitual usage Inconsistent usage Usage frequency has changed
	Support type	Learning a skill Monitoring Palliative usage
Qualities of the tool that encourage continued use	Explicitly supports goals	Being able to quantifying/visualise behaviour and consequences Feeling the app benefits them Feeling the tool is motivating Having milestones and a sense of progress Feeling and seeing accomplishments
	Feels light/not heavy	Feeling it's easy and convenient Compassionate, lack of pressure Feeling sensory pleasure/enjoyment Having choices
Conditions for sustained behaviours	Personal and contextual characteristics	Expecting difficulties High internal motivation Feeling good (reason) Influence of others
	Characteristics of the behaviour	The tool/behaviour is part of routine Behaviours in line with identity + values Behaviour feels effortless
Engagement beyond the DBCI	Efforts placed outside the use of technology	Giving themselves accountability Intentionally finding other contexts and moments to practise skills Making extra efforts (misc) Putting effort to make the behaviour a habit Self cues and reminders
	Acquired skills and abilities	Being able to regulate emotions and stress Being mindful/aware Being able to focus Not needing the tool for support New knowledge
Challenges in the process	Habit related challenges	Building a habit is a challenge Changing or too many priorities Hectic Routine
	Motivation related challenges	Having to put too much effort Mindset or mood
Positive outcomes	Positive behaviours	Increasing/modifying behaviour Spill over effect
	Positive emotions and feelings	Feeling Balance Feeling calm, relaxed Feeling good (outcome) Feeling in control

Data and Atlas.ti projects are available in the Open Science Framework (OSF) data repository: https://osf.io/yxsvw/?view_only=c25c9ba3bc07403f9c83c52ab786fc36
